# Commissioning of optical surface imaging systems for cranial frameless stereotactic radiosurgery

**DOI:** 10.1002/acm2.13240

**Published:** 2021-03-29

**Authors:** Lei Zhang, Sarath Vijayan, Sheng Huang, Yulin Song, Tianfang Li, Xiang Li, Elizabeth Hipp, Maria F. Chan, Hsiang‐Chi Kuo, Xiaoli Tang, Grace Tang, Seng Boh Lim, Dale Michael Lovelock, Ase Ballangrud, Guang Li

**Affiliations:** ^1^ Department of Medical Physics Memorial Sloan Kettering Cancer Center New York NY USA; ^2^ Department of Medical Physics Memorial Sloan Kettering Cancer Center Bergen NJ USA; ^3^ Department of Medical Physics Memorial Sloan Kettering Cancer Center Commack NY USA; ^4^ Department of Medical Physics Memorial Sloan Kettering Cancer Center Nassau NY USA; ^5^ Department of Medical Physics Memorial Sloan Kettering Cancer Center Monmouth NJ USA; ^6^ Department of Medical Physics Memorial Sloan Kettering Cancer Center Baskin Ridge NJ USA; ^7^ Department of Medical Physics Memorial Sloan Kettering Cancer Center Norwalk CT USA; ^8^ Department of Medical Physics Memorial Sloan Kettering Cancer Center Westchester NY USA

**Keywords:** calibration and commissioning, couch‐angle dependency, frameless stereotactic radiosurgery (SRS), optical surface imaging (OSI), surface‐guided frameless radiosurgery

## Abstract

**Purpose:**

This study aimed to evaluate and compare different system calibration methods from a large cohort of systems to establish a commissioning procedure for surface‐guided frameless cranial stereotactic radiosurgery (SRS) with intrafractional motion monitoring and gating. Using optical surface imaging (OSI) to guide non‐coplanar SRS treatments, the determination of OSI couch‐angle dependency, baseline drift, and gated‐delivered‐dose equivalency are essential.

**Methods:**

Eleven trained physicists evaluated 17 OSI systems at nine clinical centers within our institution. Three calibration methods were examined, including 1‐level (2D), 2‐level plate (3D) calibration for both surface image reconstruction and isocenter determination, and cube phantom calibration to assess OSI‐megavoltage (MV) isocenter concordance. After each calibration, a couch‐angle dependency error was measured as the maximum registration error within the couch rotation range. A head phantom was immobilized on the treatment couch and the isocenter was set in the middle of the brain, marked with the room lasers. An on‐site reference image was acquired at couch zero, the facial region of interest (ROI) was defined, and static verification images were captured every 10° for 0°–90° and 360°–270°. The baseline drift was assessed with real‐time monitoring of the motionless phantom over 20 min. The gated‐delivered‐dose equivalency was assessed using the electron portal imaging device and gamma test (1%/1mm) in reference to non‐gated delivery.

**Results:**

The maximum couch‐angle dependency error occurs in longitudinal and lateral directions and is reduced significantly (*P* < 0.05) from 1‐level (1.3 ± 0.4 mm) to 2‐level (0.8 ± 0.3 mm) calibration. The MV cube calibration does not further reduce the couch‐angle dependency error (0.8 ± 0.2 mm) on average. The baseline drift error plateaus at 0.3 ± 0.1 mm after 10 min. The gated‐delivered‐dose equivalency has a >98% gamma‐test passing rate.

**Conclusion:**

A commissioning method is recommended using the 3D plate calibration, which is verified by radiation isocenter and validated with couch‐angle dependency, baseline drift, and gated‐delivered‐dose equivalency tests. This method characterizes OSI uncertainties, ensuring motion‐monitoring accuracy for SRS treatments.

## 1 INTRODUCTION

Optical surface imaging (OSI) systems have been clinically applied for initial surface‐guided patient setup and intrafractional motion monitoring in frameless cranial stereotactic radiosurgery (SRS).^1^, ^2^, ^3^, ^4^, ^5^, ^6^ Before cone‐beam computed tomography (CBCT) patient setup, real‐time surface guidance allows fast, in‐room patient alignment with six degrees of freedom (DOF) using the facial area of an open‐face mask as the region of interest (ROI).^3^, ^6^, ^7^ After CBCT 6DOF couch shifts, a new OSI surface reference is captured for intrafractional motion monitoring with 0.2 mm motion detection sensitivity^3^, ^8^ and the radiation beam will be held if the motion exceeds the set tolerance.

A widely used OSI system in SRS treatment is the AlignRT system (VisionRT, London, UK) with three ceiling‐mounted camera pods (two lateral and one frontal). At any couch rotation, at least two camera pods can see the ROI.^6^ Inside each pod, two cameras provide stereoscopic views of the patient and a structured light with a speckle pattern provides identifiable point array to reconstruct a three‐dimensional (3D) surface image.^4^ The full surface image is sutured with three surfaces from the three‐camera pods that have been calibrated to the machine isocenter. As the couch rotates, the camera views change and the uncertainties of the three pods contribute differently to the system accuracy. As extremes, three camera views at couch zero reduce to two camera views at couch ±90°. Moreover, the AlignRT reference image captured at couch zero must be rotationally transformed around its isocenter to serve as the reference at SRS treatment couch angles,^3^, ^9^, ^10^, ^11^ so the uncertainty of the isocenter calibration may be amplified by the transformation.^11^ All these uncertainties matter as an SRS treatment has very tight tolerance of 1.0 mm, combining all errors from simulation to treatment.^3^, ^11^, ^12^ Therefore, any sub‐mm errors, including couch‐angle dependency and baseline drift error, need to be characterized during system commissioning.

Additionally, the performance of AlignRT beam holding through the motion management interface (MMI) with a linear accelerator (Linac) needs to be evaluated. It was reported that AlignRT position‐gating response time (latency) was >200 ms^13^, ^14^ while TrueBeam Linac has 2 ms beam‐on and 60 ms beam‐off latency.^15^ Therefore, gated‐delivered‐dose uncertainties using AlignRT should be assessed in the commissioning process and checked during routine QA procedures.^16^

In our institution, we have developed a commissioning method to characterize the AlignRT systems and quantify the optimal conditions for cranial frameless SRS treatments. Between 2011 and 2019, 17 AlignRT systems at nine clinical centers were commissioned with the couch‐angle dependency and baseline drift tests after each of the three vendor‐recommended calibration methods, that is, 1‐level plate calibration, 2‐level plate calibration, and cube calibration. A method to demonstrate the gated‐delivered‐dose equivalency was established using non‐gated plan delivery as the reference in the gamma test. This note shares the commissioning method as initial quality assurance (QA) for AlignRT‐gated SRS treatments.

## 2 MATERIALS AND METHODS

AlignRT systems provided three different imaging resolutions (high, medium, and low) that were bounded to anatomical sites, and the high resolution was used for the intracranial SRS treatment and commissioning. It images the entire field of view that the camera can see (>1 m^2^), while the ROI is small only cover the facial area. All AlignRT systems were configured in the same way across the institution as part of the SRS commissioning, including the IEC coordinate system, default clinical SRS thresholds for motion monitoring, and default user group rights to meet our clinic requirements for therapist, dosimetrist, and physicist. All participating physicists were trained for both AlignRT operations and SRS treatments.

### 2.A Clinical AlignRT systems

Seventeen AlignRT systems (v5.0/v5.1) in nine clinics across our institution were included in this study. All AlignRT systems had the plate calibration (1‐level and 2‐level) methods while only eight systems had the MV cube calibration method available. The MMI was installed to enable communication between AlignRT and the Linacs (TrueBeam or Trilogy, Varian Medical Systems, Palo Alto, CA), equipped with 6DOF couches (PerfectPitch, Varian Medical), for beam gating during SRS treatments. The large number of AlignRT systems and one uniform procedure used in this study allowed us to statistically evaluate and identify a calibration method(s) that can provide sub‐mm uncertainties in couch‐angle dependency and baseline drift and quantify the uncertainties in gated‐delivered‐dose equivalency. Eleven trained medical physicists and three physics residents participated in the experiments and data analysis.

### 2.B Three vendor‐recommended calibration methods

**Fig. 1:**
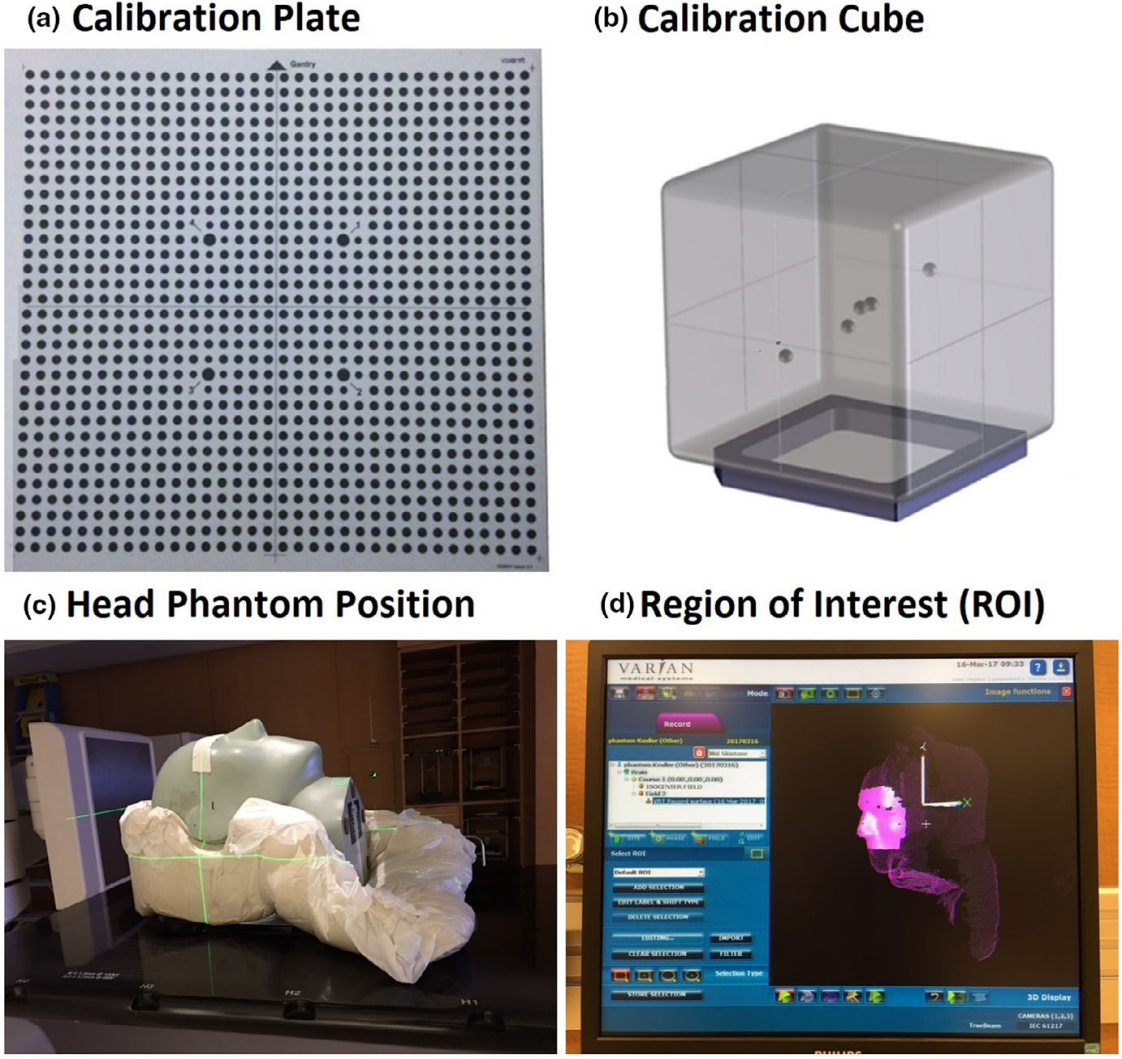
AlignRT calibration plate (a), MV cube (b), an anthropomorphic head phantom (c), and the region of interest (ROI) and isocenter position (d). The laser cross point (c) and the origin of the coordinate system (d) indicate the isocenter position in the middle of the brain. Inside the MV cube phantom, there are five ball‐bearing (bb’s) and one is placed at the center.

The calibration plate (100 × 100 cm^2^) with a dot array (34 × 34 black dots) on a white non‐reflective flat surface [Fig. 1(a)] was used for the 1‐level (2D) and 2‐level (3D) plate calibrations. The plate setup on the treatment couch was guided by room lasers, aligned with gantry crosshair projection and optical distance indicator (ODI), and verified by gantry rotation (±45°). The 1‐level plate calibration was 2D since the dot array only at the isocenter level for surface reconstruction. The 2‐level (or raised) plate calibration, however, was 3D with an additional plate level at 7.5/10.0 cm above the isocenter by raising the treatment couch. The plate calibration was used for both surface image reconstruction and isocenter establishment. In contrast, the MV cube calibration was only to verify and adjust the AlignRT isocenter. The cube phantom (15 × 15 × 15 cm^3^) with a smooth surface contained five radiopaque spheres (ϕ = 7.5 mm) for MV imaging [Fig. 1(b)]. The cube was positioned on a leveling plate and aligned to the isocenter using the crosshair and room lasers. A static 3D AlignRT image was captured and four 2D‐MV images with a field size of 10 × 10 cm^2^ were acquired at gantry angles of 270°, 0°, 90°, and 180° for AlignRT 6DOF isocenter verification and correction from the radiographic reference. The characterization of the three calibration methods was summarized in Table 1.

**Table 1:** Three vendor‐recommended calibration methods and their characteristics of AlignRT.

Category	Comparable item	Plate calibration		Cube calibration (vs MV)^#^
		1‐level	2‐level	
Calibration condition	Plate/cube positioning	Room laser and gantry crosshair	Room laser and gantry crosshair	Room laser and AlignRT surface
	Verification	±45˚ gantry rotation	±45˚ gantry rotation	Cube surface alignment
	Re‐positioning	NA	Raised plate	NA
	Plate/cube level	ISO	ISO & 7.5cm or 10.0 cm higher	ISO
Calibration characteristics	ISO calibration	Yes	Yes	Yes
	ISO accuracy	Mid to high	Mid to high	High
	Reconstruction	Yes	Yes	No
	Recon dimension	2D	3D	NA
	Recon accuracy	Mid	High	NA

^#^ Megavoltage (MV) radiation beam provides the ground truth of a Linac isocenter.

### 2.C Determination of the couch‐angle dependency error

After each calibration, the couch‐angle dependency experiment was conducted using an anthropomorphic Kessler/Steve head phantom (CIRS, Norfolk, VA), as shown in Fig. 1(c). The head phantom was immobilized in a customized mold on the treatment couch with the isocenter in the middle of the brain aligned with lasers. An on‐site static reference image was captured at couch zero and an ROI was drawn, similar to the clinical ROI with an open‐face mask [Fig. 1(d)]. The couch was rotated from 0° to ±90° with 10° interval around the isocenter and a static verification image was captured at each couch angle for registration with the rotated reference. The registration produces a shift (error) in 6DOF, indicating the deviation of the alignment from the ideal null shift. The couch‐angle dependency error was defined as the maximum registration error within the couch rotation range (0 to ±90°).

### 2.D Determination of the baseline drift error

The baseline drift error was measured using the same head phantom by applying real‐time‐delta (RTD) imaging for 20 min continuously. The turning point of the drift deviating from null before leveling off was defined as the baseline drift error. This uncertainty was originated from system heating, even with a cool light‐emitting device (LED) for sparkle light projection. In one system, the baseline drift test was repeated after the system was idled for 20 min to check the reproducibility.

### 2.E Determination of gated‐delivered‐dose equivalency with non‐gated delivery reference

When the MMI was enabled, AlignRT communicated with a Linac, triggering beam hold when RTD shifts exceeded a set threshold. A 1.0 mm threshold was used at couch zero, while a 1.5 mm threshold was used at other couch angles to compensate for the couch‐angle dependency error (usually 0.5–1.0 mm). The default display range for motion monitoring was set as 3 mm and 3° for SRS treatments. System latencies in beam‐on and beam‐off trigger between AlignRT and Linac could cause dosimetric uncertainties in treatment delivery. To investigate the overall dosimetric accuracy of a gated delivery guided by AlignRT, the gated‐delivered‐dose equivalency test was conducted using real‐time positioning management (RPM) motion phantom (Varian, Palo Alto, CA), which was set outside of the beam field and a thin plastic manikin was placed on top to allow AlignRT to monitor its motion and trigger the beam hold via the MMI communication when it moved beyond the threshold. The gated VMAT QA plan was delivered at a fixed gantry angle (0°) without rotation and was recorded by the electronic portal imaging device (EPID) and compared with no motion non‐gated reference delivery using gamma analysis with three different criteria: 3%/2 mm for SRT and 2%/1 mm and 1%/1 mm for SRS. The Varian EPID dosimetry software was used for the local gamma test with a minimum threshold of 10%.

## 3 RESULTS

**Fig. 2:**
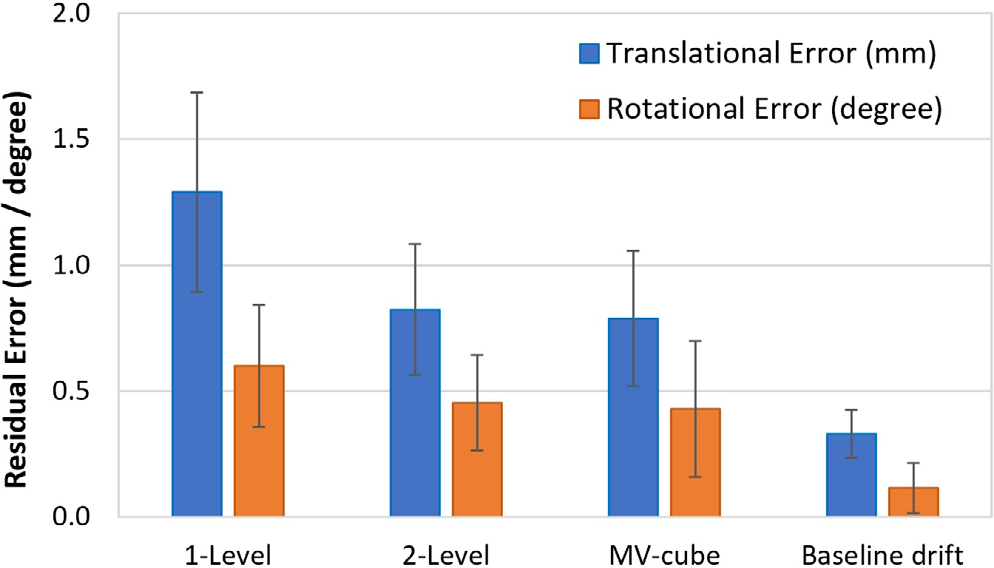
Couch‐angle dependency (CAD) error after each calibration (1‐level plate calibration, 2‐level plate calibration, and MV cube calibration) and the baseline drift (BLD) error. Average translational (blue) and rotational (orange) errors with one standard deviation are shown.

**Fig. 3:**
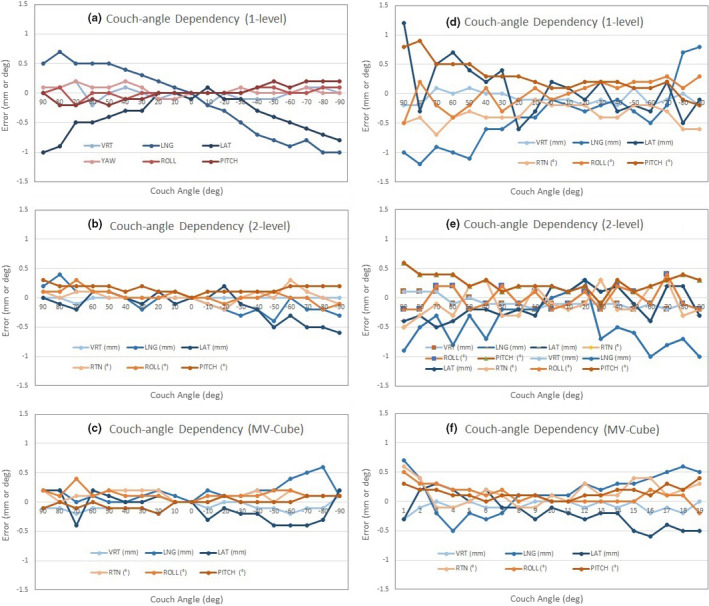
Two typical AlignRT calibration examples (1: Left and 2: Right) of the couch‐angle dependency (CAD) error after the 1‐level plate calibration (a, d), 2‐level plate calibration (b, e), and MV cube calibration (c, f). The longitudinal (LNG) and lateral (LAT) produce the biggest CAD errors. In calibration example 1, the data were from 0° → ±90° couch rotation, while in calibration example 2, the data were from 0°← ±90° couch rotation, so that a slight uncertainty (~0.2 mm and 0.2°) may be found when the couch returned to zero.

**Fig. 4:**
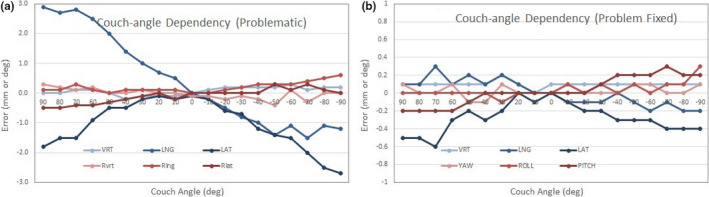
An example of problematic 1‐level calibration: before (a) and after (b) fixing the problem. The miscalibration problem (a) was simulated with on‐purpose misplacement of the plate in horizontal directions by 1–2 mm, followed by an accurate plate placement for calibration.

Results of couch‐angle dependency after each calibration method and the baseline drift of the systems are summarized in Fig. 2. There is a significant reduction in the couch‐angle dependency error (*P* < 0.05) using the 2‐level plate calibration (0.8 ± 0.3 mm) compared to 1‐level plate calibration (1.3 ± 0.4 mm), illustrating the improved accuracy of 3D calibration over 2D calibration. Along each of the six axes (translational and rotational), the couch‐angle dependency errors are significantly different (*P* < 0.05, Table 2), and the errors in longitudinal and lateral directions near ±90° (>50°) are the largest, as shown in Fig. 3. The result is not affected by changing the direction of couch rotation although hysteresis was observed within 0.2 mm. The couch‐angle dependency error after MV cube calibration is similar (0.8 ± 0.2 mm) to that from 2‐level calibration. Figure 4 illustrates an example of catching calibration error using this couch‐angle dependency test in troubleshooting.

**Table 2:** Comparison of the distribution of couch‐angle dependency (CAD) errors in six degrees of freedom (DOF) between 1‐level and 2‐level plate calibrations. The major errors and error reductions are in the longitudinal (LNG) and lateral (LAT) directions, while all CAD errors are reduced significantly after using 2‐level calibration.

System	CAD error after 1‐level calibration							CAD error after 2‐level calibration						
	Translation error (mm)				Rotation error (˚)			Translation error (mm)				Rotation error (˚)		
	VRT	LNG	LAT	MAG	RTN	ROLL	PITCH	VRT	LNG	LAT	MAG	RTN	ROLL	PITCH
1	‐	‐	‐	‐	‐	‐	‐	0.2	0.6	0.8	1.0	0.1	0.2	0.2
2	0.2	0.5	0.6	0.8	0.3	0.3	0.3	0.2	0.2	0.4	0.4	0.1	0.4	0.3
3	0.2	0.6	0.4	0.7	0.2	0.4	0.2	0.2	0.6	0.4	0.7	0.2	0.4	0.2
4	0.2	2.0	1.2	2.3	0.9	0.4	0.6	0.1	0.9	0.6	1.1	0.1	0.1	0.2
5	0.1	0.8	0.8	1.1	0.5	0.3	0.3	0.2	0.6	0.7	0.9	0.4	0.3	0.2
6	0.1	1.3	0.8	1.5	0.6	0.2	0.1	0.1	0.4	0.5	0.6	0.0	0.2	0.2
7	0.1	0.8	0.8	1.1	0.5	0.6	0.5	0.2	0.5	0.7	0.9	0.2	0.3	0.3
8	0.2	0.8	1.4	1.6	0.4	0.2	0.3	0.1	0.8	0.8	1.1	0.2	0.1	0.1
9	0.2	1.0	0.5	1.1	0.3	0.2	0.1	0.1	0.8	0.8	1.1	0.2	0.1	0.1
10	0.1	1.3	1.4	1.9	0.2	0.4	0.3	0.2	0.3	1.1	1.1	0.5	0.2	0.3
11	0.7	1.2	1.5	2.0	0.9	0.4	0.8	0.5	1.4	1.1	1.8	0.4	0.4	0.6
12	0.2	1.2	1.2	1.7	0.7	0.5	0.9	0.2	1.0	0.5	1.1	0.5	0.4	0.6
13	0.3	0.6	1.9	2.0	0.9	0.4	0.6	0.2	0.4	0.8	0.9	0.9	0.4	0.6
14	0.6	1.4	0.6	1.6	0.6	0.4	0.4	0.2	0.6	0.5	0.8	0.3	0.3	0.3
15	0.3	1.3	1.4	1.9	0.9	0.7	0.4	0.3	0.9	0.9	1.3	0.6	0.4	0.4
16	0.5	1.1	0.7	1.4	0.6	0.4	0.4	0.2	0.6	0.4	0.7	0.3	0.2	0.2
17	0.8	1.6	1.3	2.2	0.5	0.3	0.5	0.5	0.7	0.8	1.1	0.4	0.1	0.3
MEAN	0.3	1.1	1.0	1.6	0.6	0.4	0.4	0.2	0.7	0.7	1.0	0.3	0.3	0.3
SD	0.2	0.4	0.4	0.5	0.2	0.1	0.2	0.1	0.3	0.2	0.3	0.2	0.1	0.2
*P*‐value*								0.05	0.001	0.001	0.000	0.001	0.003	0.005

* The p‐value is from the Student t‐test of the corresponding columns of the CAD errors between 1‐level and 2‐level calibration. The differences in 6DOF are significant (*P* < 0.05).

**Fig. 5:**
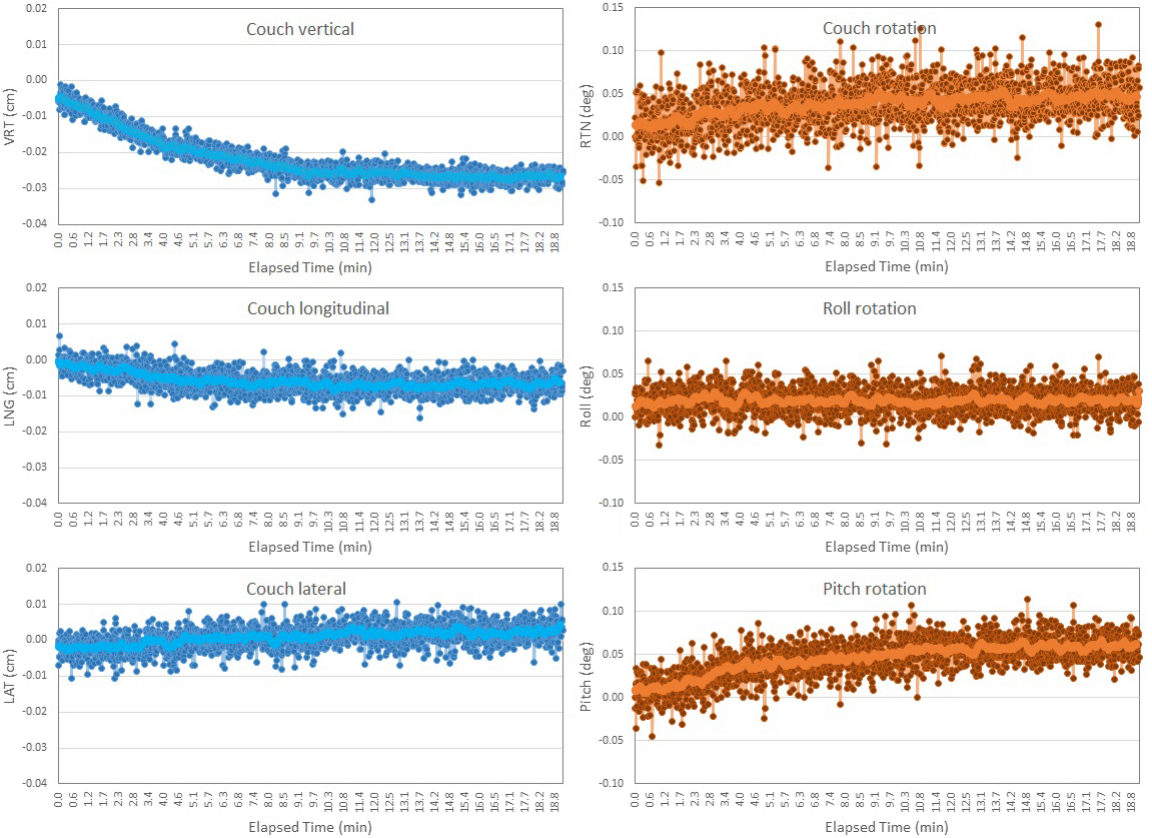
An example of the baseline drift (BLD) error in 6 degrees of freedom (DOF) that leveled off after 10 min continuous real‐time delta (RTD) motion monitoring. The largest drift occurs in the vertical direction. The light‐colored lines are the trend lines (moving average).

Figure 5 shows that the baseline drift, most obvious in the vertical direction, levels off at 0.3 mm after about 10 min of RTD monitoring, and the same trend was observed across the systems included in this study. The baseline drift results are reproducible after 20 min cooling off. The baseline drift has been reduced by ~50% in the latest high‐definition camera system compared with the original camera system.

Table 3 illustrates the gated‐delivered‐dose equivalency using the EPID portal dosimetry measurement and the gamma test (local gamma with 10% minimum threshold) using the no motion non‐gated delivery as the reference. The average gamma pass rate (±σ) is 99.8 ± 0.4% with 3%/2 mm for SRT, and 99.0 ± 1.4% with 2%/1 mm and 98.3 ± 1.7% with 1%/1 mm for SRS.

**Table 3:** Gated dosimetry equivalency in reference to non‐gated delivery using the electronic portal imaging device (EPID) for portal dosimetry measurement.

AlignRT system	Linac system	Gamma test criteria		
		3%/2 mm	2%/1 mm	1%/1 mm
1	TrueBeam	100.0	100.0	100.0
2	TrueBeam	100.0	100.0	98.0
3	TrueBeam	100.0	99.5	98.5
4	TrueBeam	100.0	99.9	98.5
5	TrueBeam	100.0	100.0	100.0
6	TrueBeam	100.0	100.0	100.0
7	TrueBeam	100.0	100.0	99.0
8	Trilogy	99.2	97.2	96.5
9	Trilogy	99.7	97.1	95.4
10	TrueBeam	100.0	100.0	100.0
11	TrueBeam	99.6	98.0	97.4
12	TrueBeam	100.0	98.7	98.2
13	TrueBeam	100.0	100.0	99.9
14	TrueBeam	100.0	100.0	100.0
15	TrueBeam	100.0	99.4	98.5
16	TrueBeam	99.7	97.4	95.6
17	Trilogy	98.4	96.2	95.2
Average		99.8	99.0	98.3
St Dev		0.4	1.3	1.7

## 4 DISCUSSION

### 5.A AlignRT system commissioning for cranial SRS treatments

This commissioning study provides the largest clinical study on couch‐angle dependency, baseline drift, and gated‐delivered‐dose uncertainties on 17 AlignRT systems. The couch‐angle dependency error is attributed to limited number of camera pods, uncertainties in 2D/3D calibrations and isocenter determination, and Linac couch walkout. The three‐camera system is the minimum requirement for SRS as they provide minimal views necessary at all couch angles. The average couch‐angle dependency errors of 1.6 ± 0.5 mm and 0.6° ± 0.2° using 1‐level plate calibration over the 17 AlignRT systems are similar to a previous report, in which the phantom data showed a mean (max) error of 1.2 (2.3) mm and 0.7°(1.2°) at the couch angles of ±90° in one AlignRT system.^2^ For SRS treatments, the error tolerance is sub‐mm, which is much tighter than other anatomical sites, such as breast treatments.^17^ By using the 2‐level plate calibration, the average errors are reduced to 1.0 ± 0.3 mm and 0.3° ± 0.2°, as shown in Table 2. Our results showed that the improvement in 3D surface reconstruction using the 2‐level plate calibration reduces the couch‐angle dependency error compared to the 1‐level calibration, because the vertical dimension data are incorporated into the reconstruction and the raised plate level is closer to the phantom surface. Therefore, the 3D plate calibration with MV isocenter verification is recommended, which can be achieved by either 2‐level plate calibration or Advanced Camera Optimization (ACO) calibration, performed by the vendor’s annual service. Note that ACO is to use a 3D optical calibration model to enhance the accuracy and stability of the system.^18^ The AlignRT couch‐angle dependency error has been evaluated for both isocentric Linacs^11^, ^18^ and robotic Linac.^19^

The benefits of MV cube isocenter calibration is not obvious from our results, and it is mainly dependent on the quality of plate calibration, namely the plate setup accuracy. Proper plate alignment using the gantry crosshair projection with ±45° gantry rotations is warranted to ensure the accuracy of plate calibration, since room laser and ODI systems can carry a 0.5‐mm uncertainty.^20^ Couch walkout also contributes to a small deviation (~0.5 mm for Trilogy and 0.1–0.5 mm for TrueBeam) between the mechanical and radiation isocenters that eventually are convoluted in the total couch‐angle dependency error. After all, the couch‐angle dependency error is difficult to correct but can be minimized to within 1.0 mm. Previously, we have compared the AlignRT accuracy with 2DkV and CBCT using a system that does not have MV cube calibration capability,^21^ and the sub‐mm differences are consistent with the MV cube calibration.

The baseline drift error due to camera heating is relatively small (0.3 mm).^3^ It is mostly manifested along the vertical axis while couch‐angle dependency errors are more significant in longitudinal and lateral directions. To minimize the baseline drift error, it is important to use RTD during the initial AlignRT and CBCT setup to allow the system to reach a steady‐state stage before treatment. This can not only warm up the system but also provide patient monitoring during CBCT before treatment. Both couch‐angle dependency and baseline drift tests are considered necessary for AlignRT commissioning, with exception of fixed couch‐angle Linac system, such as Halcyon system,^22^ in which only baseline drift test is necessary.

If the MMI communication is enabled between AlignRT and Linac, the gated‐delivered‐dose equivalency test should be included as part of AlignRT commissioning. Our result indicates that the difference between gated and non‐gated plan delivery is negligible, even under the tightest gamma test criteria (1%/1 mm). This dose equivalency test is important in identifying AlignRT induced gating errors that is usually not included as part of an end‐to‐end test.^5^, ^23^ In addition, the result of gated‐delivered‐dose equivalency suggests that the AlignRT‐Linac system response latency^13^, ^14^, ^15^ may be negligible for brain SRS treatment with a frameless immobilization system.^3^, ^6^

### 5.B System configuration and commission statistics from 17 AlignRT systems

AlignRT provides three (high, medium, and low) imaging resolutions, based on anatomical sites. It is essential to select the highest OSI resolution for SRS commissioning and treatment, and the high‐resolution anatomies are intracranial SRS, brain, face, and neck. AlignRT static capture provides higher image resolution with the full field of view, while the dynamic RTD provides real‐time imaging at 4Hz with slightly lower resolution focusing on the ROI only.

In the MV cube calibration, the AlignRT isocenter was calibrated against the MV radiation isocenter of an Linac using a test similar to the Winston–Lutz test. Therefore, the commissioning of AlignRT systems is sufficiently verified for clinical use. More importantly, in the image‐guided radiotherapy (IGRT) era, the room laser system used in the Winston–Lutz test is no longer used as a reliable surrogate for tumor localization. In contrast, the validation of imaging isocenter vs radiation isocenter is the key to ensure IGRT treatment accuracy. In the MV cube calibration, the AlignRT isocenter is validated against the MV isocenter. In daily clinical SRS treatments, 2DkV and CBCT imaging isocenters were validated by acquiring both kV and MV 2D or 3D images for alignment.

It is important to have the statistics from the commissioning data of 17 AlignRT systems, which include the technical differences among the AlignRT systems and the Linac systems, such as couch walkout. Although the individual difference of the physicists who performed the commissioning may also play a role in the statistics, it would be a relatively small factor to concern in our institution. Among the 17 systems, different Linac systems would have a slight variation of the couch walkouts, which are embedded in the couch‐angle dependency errors. This is true that the Trilogy systems produce higher uncertainties than the TrueBeam systems due to the differences in their isocenter accuracy of the couch. In our Trilogy Linacs, the couch walkout can be >0.5 mm while TrueBeam Linacs’ couch walkout is generally <0.5 mm.

### 5.C Clinical benefits of the commissioning in SRS treatment

The direct clinical benefit of the AlignRT commissioning procedure contains multiple aspects. First, it is helpful to determine the clinical action threshold for patient motion monitoring during SRS treatment. As the couch‐angle dependency error is 0.5–1.0 mm, we set the action threshold at 1.0 mm at couch zero and 1.5 mm for non‐coplanar beams/arcs in our clinics; otherwise, the SRS treatment would be interrupted by many >1.0 mm false positives in motion detection. A 1˚ tolerance is used for all couch angles. This is especially true when treating SRS with a VMAT plan that involves gantry rotation, which can block one of the AlignRT camera pods, resulting in an up to 1 mm additional uncertainty.^24^ Second, as we learn the degree of baseline drift, we apply RTD not only at in‐room patient alignment using AlignRT guidance but also during CBCT scanning, registration, and setup approval by attending physician. By the time that treatment starts, the AlignRT camera system has been warmed up in RTD mode for more than 5 min. Therefore, the baseline drift error can be minimized. Third, the gated‐delivered‐dose equivalency ensures that occasional beam hold triggered by temporary head motion exceeding the threshold will not significantly affect the delivered radiation dose to the brain target.

Based on our experience, a 6DOF couch is highly desirable to minimize rotational error at the setup since the rotation error may be tangled with translation error, causing additional uncertainties. The couch‐angle dependency test can also be applied to clinical troubleshooting for accidental miscalibration, if the false‐positive incidence level of SRS patient motion is raised, as demonstrated in Fig. 4. If the MV cube calibration is not available in a clinic, the couch‐angle dependency test would be the alternative method to perform during system calibration to ensure accurate and smooth SRS treatments.

In addition, because of the fast in‐room patient alignment using AlignRT guidance in 6DOF, the patient is very close to the final setup position at the time of CBCT scan. Therefore, after CBCT, only <2 mm and 1° couch shifts may be needed, eliminating the need for additional kV or CBCT imaging verification. Therefore, both patient setup time and imaging radiation dose are substantially reduced. A similar observation was also reported.^25^ Because of the advantage of one CBCT per patient setup, since late 2016 our clinic has further applied the SRS treatment procedure to treat hypo‐fraction brain SRT patients with reduced treatment margin of 2 mm and faster surface/image‐guided patient setup. Overall, based on our clinical experience, using the AlignRT commissioning procedure ensures the safety and smoothness of frameless SRS and SRT cranial patient treatments. Together with other advancements in SRS treatment, including VMAT SRS planning for multi‐lesion treatment using single isocenter^21^, the SGRT SRS/SRT procedure has allowed us to treat substantially more cranial lesions and patients as the standard of care in our clinic.

## 5 CONCLUSION

We reported a large‐scale quantitative evaluation of the couch‐angle dependency, baseline drift, and gated‐delivered‐dose uncertainties from 17 AlignRT systems, which has guided our establishment of commissioning and routine quality assurance procedures of using AlignRT for cranial frameless SRS treatment. The 3D plate calibration method with MV isocenter verification is recommended for commissioning and routine calibration, because it improves 3D surface reconstruction accuracy and reduced the couch‐angle dependency error. If the MV cube calibration is not available, performing the couch‐angle dependency test would be the alternative to ensure accurate and smooth SRS treatments. The baseline drift of the system and the gated‐delivered‐dose equivalency are also important commissioning components as they affect the overall accuracy of SRS treatments. This commissioning procedure provides a quantitative evaluation of an AlignRT system for SRS treatment and the performance baseline for routine QA.
